# ETS like-1 protein ELK1-induced lncRNA LINC01638 accelerates the progression of papillary thyroid cancer by regulating Axin2 through Wnt/β-catenin signaling pathway

**DOI:** 10.1080/21655979.2021.1935404

**Published:** 2021-07-19

**Authors:** Pin Lv, Yuan Xue

**Affiliations:** The General Surgery Department, The Second Hospital of the University of ShanXi, Taiyuan, Shanxi Province, China

**Keywords:** Papillary thyroid cancer, LINC01638, Wnt/β-catenin, Axin2

## Abstract

Papillary thyroid carcinoma (PTC) characterized by distant metastasis is a major public health issue among women worldwide. LncRNA LINC01638 is reportedly a critical oncogene in the development of certain cancers. However, the biological function of LINC01638 in PTC is currently unclear. The goal of this study was to identify LINC01638 expression level and its role in PTC progression. The expression of LINC01638 was detected applying qRT-PCR. CCK-8 assay, colony formation assay, immunofluorescence staining and flow cytometric analysis were performed to assess cell proliferation and cell cycle. In addition, cell migration and invasion were examined via wound healing assay, transwell assay and western blot analysis. We found that LINC0163 was upregulated in PTC cells compared with normal thyroid gland epithelial cell line Nthy-ori3-1. ELK1 could act as a transcription factor of LINC01638 and induce LINC01638 expression. LINC01638 silencing inhibited cell proliferation, migration and invasion, and obstructed the progress of TPC-1 cell cycle. LINC0163 silencing activated Axin2 while suppressing the expressions of β-catenin, Cyclin-D1 and c-MYC. Rescue experiment utilizing the transfection of Axin2 overexpression plasmid weakened LINC01638 overexpression-enhanced TPC-1 cell proliferation, metastasis, cell cycle progress and Wnt/β-catenin pathway. These results indicate that LINC0163 regulates PTC progression via inhibition of Wnt/β-catenin and activation of Axin2, which may develop into a novel therapeutic strategy for PTC treatment.

## Graphical Abstract

**Figure:**
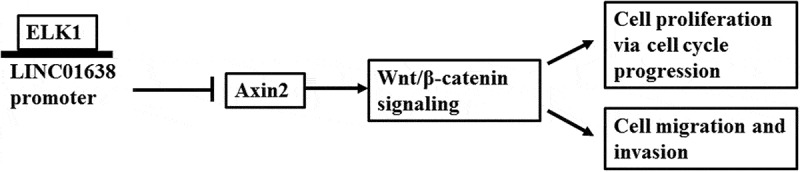

## Introduction

Papillary thyroid carcinoma (PTC), from a histological perspective, is the most common category of thyroid cancer (TC), but with a relatively low level of malignancy [1,2]. The 5-year survival of PTC can be roughly as high as 95%, but its feature of distant metastases to tissues and lymph nodes of PTCs with more aggressive phenotypes promote it to a more lethal thyroid cancer, and the survival rate can thus be reduced to 50% [3]. Although much advance has been made on diagnose and therapy of PTC, the curative effect remains disappointing [4]. Conventional treatment strategies for PTC, such as surgery, chemotherapy and radioiodine therapy, are currently futile in patients with metastatic PTC [5]. Therefore, it is urgent to discover novel therapeutic alternatives and treatment strategies for patients with metastatic PTC and lethal thyroid cancers.

Long non-coding RNAs (lncRNAs), referring to non-coding RNAs greater than 200 nucleotides in length, are not equipped with protein-encoding abilities but are able to regulate the transcriptional, epigenetic and posttranscriptional process of gene expression [6,7]. It has been verified that many lncRNAs play fatal roles in multiple physiological activities of human cancers [8–10]. The connections of many lncRNAs to PTC development have been determined by extensive medical research [11]. LncRNA LINC01638 has shown oncogenic effects in several tumors. Zhuo et al. revealed that LINC01638 expression was obviously increased in colorectal adenocarcinoma patients while LINC01638 silencing repressed cell proliferation of colorectal adenocarcinoma through interaction with RUNX2 [12]. Another study discovered that LINC01638 was overexpressed in the tissues and cells of triple-negative breast cancer (TNBC) and that its depletion largely prevented TNBC cell proliferation and invasion through regulation of MTDH-Twist1 signaling pathway [13]. Despite what is mentioned, the functional role of LINC01638 in PTC is still awaiting a definition.

Therefore, the present study aims to explore the role of LINC01638 in PTC and attempt to clarify its molecular mechanism in which LINC01638 affects the occurrence and development of PTC. The results of the current study revealed that ELK1 and LINC01638 level was upregulated in PTC cells. ELK1 acted as a transcription factor of LINC01638. LINC01638 knockdown inhibited cell proliferation, metastases and invasion and blocked cell cycle progression by regulating Axin2 through Wnt/β-catenin signaling pathway. This study provides a potential novel biomarker or therapeutic strategy for PTC treatment.

## Materials and methods

### Cell culture

Thyroid gland epithelial cell line Nthy-ori3-1 and PTC cell lines TPC-1, IHH-4 and BCPAP were purchased from CCTCC (China Center for Type Culture Collection, Shanghai, China). These cells were cultured with RPMI 1640 medium (Invitrogen; Thermo Fisher Scientific, Inc., Waltham, MA, USA) supplemented with 10% fetal bovine serum (FBS) at 37°C in an atmosphere of humidified air containing 5% CO_2_.

### Cell transfection

The short hairpin RNA (shRNA)-LINC01638-1/2, overexpression plasmid of LINC01638 (pcDNA-LINC01638) and Axin2 (Axin2 OV), as well as their corresponding negative controls negative control groups were synthesized by GenePharma (Shanghai, China). The plasmid vectors were transfected into TPC-1 cells, respectively, with Lipofectamine 2000 reagent (Invitrogen) following the manufacturer’s instructions. At 48 h post-transfection, the transfected cells were collected for the subsequent experiments.

### Quantitative real-time PCR (qRT-PCR)

Isolation of the total RNA in PTC cells was conducted by means of utilizing TRIzol reagent (Invitrogen, USA) under the guidance of the product manual. Reverse transcription (RT) to form the first-strand cDNAs was performed via the utilization of PrimeScriptRT reagent kit (TAKARA, Japan). qRT-PCR reactions were conducted with SYBR Prime Script RT-PCR kit (TAKARA, Japan). The following thermocycling conditions were used for qRT-PCR: initial denaturation at 95°C for 5 min; followed by 30 cycles of 95°C for 40 sec, 57°C for 40 sec and 72°C for 40 sec, with a final extension at 72°C for 10 min. Expression levels were quantified using the 2^−ΔΔCt^ method [14]. GAPDH served as the internal control for the normalization of gene expression statistics. The primer sequences in this assay are listed as follows: LINC01638 forward, 5´-AATACATCAGCACTGTTGCCTTT-3´and reverse, 5´-CTCCATACATACATCTCCAAAAAGT-3´; Axin2 forward, 5´-CAAGGGCCAGGTCACCAA-3´ and reverse,5´-CCCCCAACCCATCTTCGT-3´;GAPDH forward, 5´-GTGGACATCCGCAAAGAC-3´ and reverse, 5´-AAAGGGTGTAACGCAACTA-3´.

### Cell counting kit-8 assay

The testing of cell viability was carried out using cell counting kit-8 (CCK-8; Dojindo, Japan). TPC-1 cells were seeded in 96-well plates (2 × 10^3^ cells/well) and cultured at 37°C for 24, 48 and 72 h after transfection. A microplate reader was used to measure the absorbance at 450 nm after 2 h of incubation with CCK-8 solution. This assay included five replications of each group.

### Colony formation assay

Cells were added into 6-well plates and cultured in DMEM with 10% FBS at 37°C. After incubation for 14 days, the cells were fixed in 4% paraformaldehyde and stained with 0.1% crystal violet reagent. Cell colony numbers were counted under a light microscope. Three replicated assays were conducted independently.

### Immunofluorescence assay

TPC-1 cell slides were fixed with 4% paraformaldehyde for 20 min and incubated with 5% skimmed milk in 0.01 M PBS at room temperature for 1 h. Anti-Ki67 (Abcam) and Alexa Fluor conjugated secondary antibodies (Life Technologies) were successively taken to incubate the slides. The slides were then washed with PBS and examined with a laser confocal microscope.

### Wound healing assay

The migration ability of TPC-1 cells subjected to different treatments was looked at through a wound healing assay. Cells seeded in 6-well plates were grown until 80% to 90% confluence. Then, the cell monolayers were wounded by using a 20-μL tip and washed three times with serum-free medium to remove cell debris. After 24 h incubation, migrated cells were observed and counted under an inverted microscopy.

### Cell invasion assay

Cell invasion was detected by an invasion assay with using transwell chamber (8 µm pore-size, Corning Costar, Cambridge, MA). The chambers were first coated with 0.1 mL of matrigel for 1 h at 37°C. Transfected cells were trypsinized and suspended in DMEM containing 1% FBS. Cell suspension was added to the upper compartment, and 10% FBS in culture medium was added to the lower one. After 24 h incubation, cells in the upper chamber were wiped off, and the invaded cells in the lower surface were fixed and stained with hematoxylin and eosin. Finally, stained cells in five randomly chosen visual fields were counted, and the pictures of each group were taken by the use of a light microscope.

### Flow cytometric analysis

The effects of LINC01638 and Axin2 overexpression on PTC cell cycle progression were detected by flow cytometric analysis. Briefly, TPC-1 cells that underwent trypsinization were harvested and fixed with 70% cold ethanol. Different cell cycle stages were observed through the propidium iodide (PI) staining technique incorporating RNase, followed by analysis of cell cycle in Flowjo software (Tree Star, Ashland, OR, USA).

### Western blot analysis

Total proteins from transfected TPC-1 cells were extracted with RIPA lysis buffer. Protein concentration was detected by a BCA Protein Assay Reagent Kit (Beyotime). Subsequently, SDS-PAGE-separated protein samples were transferred onto a PVDF membrane and then blocked in 5% nonfat milk for 2 h at room temperature. The membranes were incubated with primary antibodies against MMP2 (cat. no. ab92536), MMP9 (cat. no. ab76003), c-MYC (cat. no. ab32072), Axin2 (cat. no. ab109307), β-catenin (cat. no. ab223075), Cyclin-D1 (cat. no. ab16663) and GAPDH (cat. no. 8245; all from Abcam) at 4°C overnight and later with horseradish peroxidase-conjugated secondary antibodies for 2 h at room temperature. The bands were visualized by an enhanced chemiluminescence (ECL) reagent kit (Amersham Biosciences, USA) and analyzed by Image J (NIH, USA).

## Statistical analysis

Data were presented as the mean ± SD of at least three independent experiments and analyzed by SPSS.20 (SPSS Inc., USA). One-way analysis of variance analysis (ANOVA) was used to compare the results of multiple groups. Comparisons between the results of two groups were performed with a Student’s *t*-test. P < 0.05 is the parameter that confirms the significance of the statistical difference.

## Results

Here, we aimed to explore the role of LINC01638 in PTC and to clarify its molecular mechanism in which LINC01638 affects the development of PTC. We conducted a series of in vitro assays, and found that ELK1-induced upregulation of LINC01638 promoted cell proliferation, metastases and invasion and blocked cell cycle progression by regulating Axin2 through Wnt/β-catenin signaling pathway, accelerating PTC progression. This study for the first investigated the functional roles of LINC01638 in PTC, providing new insights into the pathogenesis of PTC.

### ELK1 induced LINC01638 in papillary thyroid carcinoma cell lines

**Figure 1.**
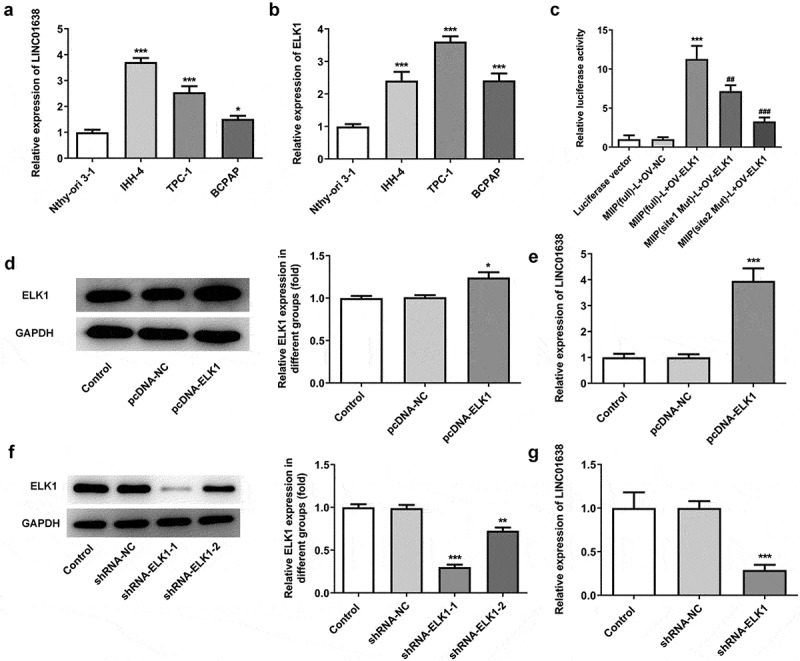
ELK1 induced LINC01638 in papillary thyroid carcinoma cell lines. A, The expression of LINC01638 in PTC cell lines TPC-1, IHH-4, BCPAP and thyroid gland epithelial cell line Nthy-ori3-1 was detected by qRT-PCR. B, The expression of LINC01638 in PTC cell lines and Nthy-ori3-1 was detected by qRT-PCR. Data are expressed as mean ± SD. **P < 0.01, ***P < 0.001 versus Nthy-ori3-1 cell line. C, The luciferase reporter assay was used to determine the binding relationship between ELK1 and LINC01638. Data are expressed as mean ± SD. ***P < 0.001 versus MIIP (full)-L+ OV-NC; ^##^P < 0.01, ^###^P < 0.001 versus MIIP (full)-L+ OV-ELK1. D, The protein expression of ELK1 was detected by western blot after transfection of pcDNA-ELK1. E, The expression of LINC01638 was detected by qRT-PCR after transfection of pcDNA-ELK1. Data are expressed as mean ± SD. *P < 0.05, ***P < 0.001 versus pcDNA-NC. F, The protein expression of ELK1 was detected by western blot after transfection of shRNA-ELK1. G, The expression of LINC01638 was detected by qRT-PCR after transfection of shRNA-ELK1. Data are expressed as mean ± SD. **P < 0.01, ***P < 0.001 versus shRNA-NC

Through JASPAR software prediction, we found that ELK1 was one transcription factor of LINC01638. To explore the role of ELK1 and LINC01638 in papillary thyroid carcinoma, we firstly looked at their expression in TPC-1, IHH-4, BCPAP and Nthy-ori 3–1. The results revealed that the expression levels of LINC01638 and ELK1 were obviously increased compared with the normal thyroid gland epithelial cells (Figure 1a-b), reflecting that both of LINC01638 and ELK1 were upregulated in PTC. Due to the highest expression of ELK1 and the relatively high expression of LINC01638 in TPC-1 cells, TPC-1 was used for the subsequent experiments. To further explore the interaction between LINC01638 and ELK1, luciferase reporter assay was conducted, and the results exhibited that the promoter activity of LINC01638 was significantly increased when ELK1 was overexpressed and was decreased when the predicted binding sequence was mutational (Figure 1c), indicating that ELK1 could induce promoter activity of LINC01638. In addition, the expression level of LINC01638 was significantly upregulated when ELK1 was overexpressed and the expression level of LINC01638 was significantly downregulated when ELK1 was knocked down in TPC-1 cells (Figure 1d-g). The data indicated that ELK1 induced LINC01638 promoter activity and the expression level of LINC01638 was regulated by ELK1.

### Knockdown of LINC01638 suppressed TPC-1 cell proliferation

**Figure 2.**
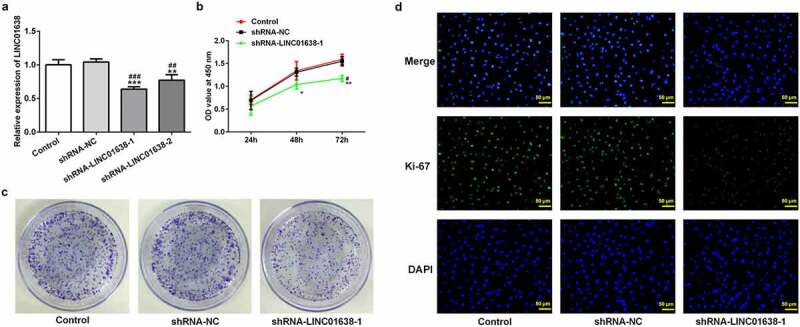
The effects of LINC01638 silencing on cell proliferation of PTC cells. A, The mRNA expression levels of LINC01638 were measured by qRT-PCR. B, CCK-8 was used to assess the cell proliferative capacity after transfection of shRNA-LINC01638. C, Colony formation assay was carried out to detect the cell colony ability. D, The level of Ki67 in TPC-1 cells was evaluated by immunofluorescence assay. Data are expressed as mean ± SD. *P < 0.05, **P < 0.01, ***P < 0.001 versus control; ^#^P < 0.05, ^##^P < 0.01, ^###^P < 0.001 versus shRNA-NC group

To identify the effect of LINC01638 silencing in PTC cells, LINC01638 shRNA vectors and negative control were transfected into TPC-1 cells. qRT-PCR was performed to assess LINC01638 expression in transfected TPC-1 cells (Figure 2a). As shown in Figure 2b, LINC01638 silencing significantly inhibited cell proliferative capacity when compared with the controls in TPC-1 cells. Additionally, colony formation assay result showed that transfection with shRNA-LINC01638 reduced cell colony number in TPC-1 cells (Figure 2c). The result from immunofluorescence assay revealed that the level of Ki67 in TPC-1 cells transfected with shRNA-LINC01638 was remarkably decreased compared with the control and shRNA-NC groups (Figure 2d). These results suggested that the low expression of LINC01638 was involved in PTC cell proliferation.

### LINC01638 silencing regulated migration and invasion of TPC-1 cells

**Figure 3.**
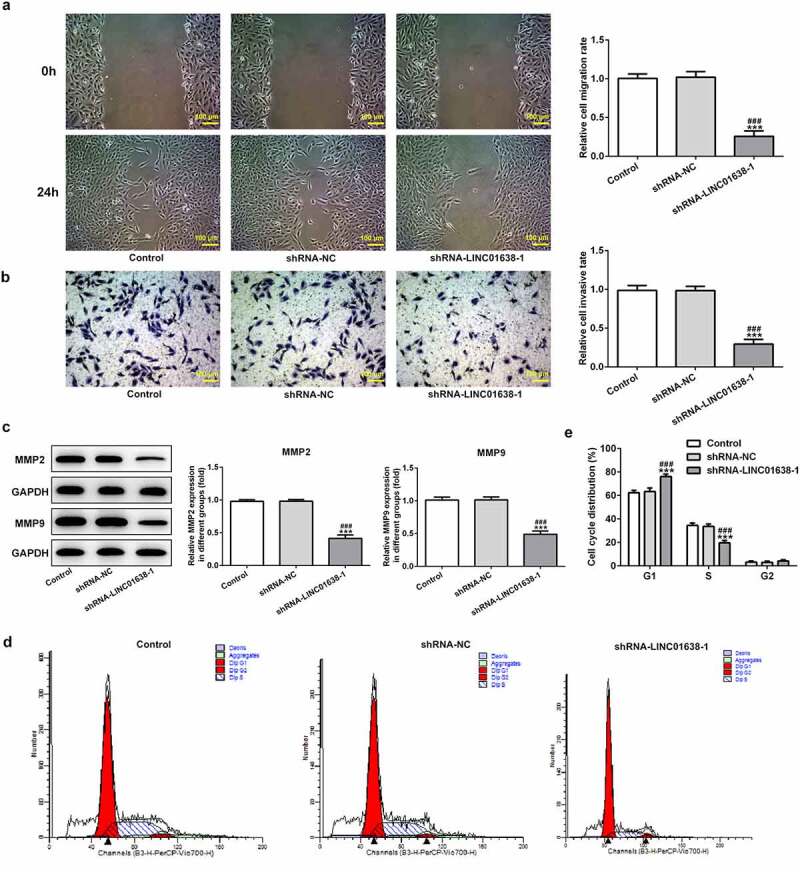
LINC01638 silencing suppresses PTC cell migration, invasion and cell cycle progression. A, Wound healing assay was performed to determine the migration of TPC-1 cells. B, Cell invasion of TPC-1 cells transfected with shRNA-LINC01638 was investigated by transwell assay. C, The effects of LINC01638 silencing on MMP2 and MMP9 were measured by western blot analysis. D and E, The cell cycle stage distribution was estimated by flow cytometric analysis. Data are expressed as mean ± SD. ***P < 0.001 versus control; ^###^P < 0.001 versus shRNA-NC group

Next, cell migration and invasion were watched following the knockdown of LINC01638 in TPC-1 cells. It can be seen from the results shown in Figure 3a and 3b that the capacity of migration and invasion in TPC-1 cells was extremely limited by knockdown of LINC01638. Furthermore, results of western blot analysis showed that LINC01638 silencing repressed the protein expression of MMP2 in transfected TPC-1 cells, as well as MMP9 (Figure 3c). These results support that LINC01638 silencing regulates migration and invasion of TPC-1 cells.

### LINC01638 silencing promoted the cell cycle blockage

The changes of cell cycle after LINC01638 knockdown were evaluated in further exploration with flow cytometric analysis. Based on the data in Figure 3d and 3e, it can be found that knockdown of LINC01638 in TPC-1 cells markedly increased the fractions of cells in S phase while reduced the percentage of G1 phase cells compared with the control and negative control groups. These data suggested that LINC01638 silencing may regulate PTC progression via altering the cell cycle stage distribution.

### The effects of LINC01638 on the proteins levels of Wnt/β-catenin pathway in TPC-1 cells

**Figure 4.**
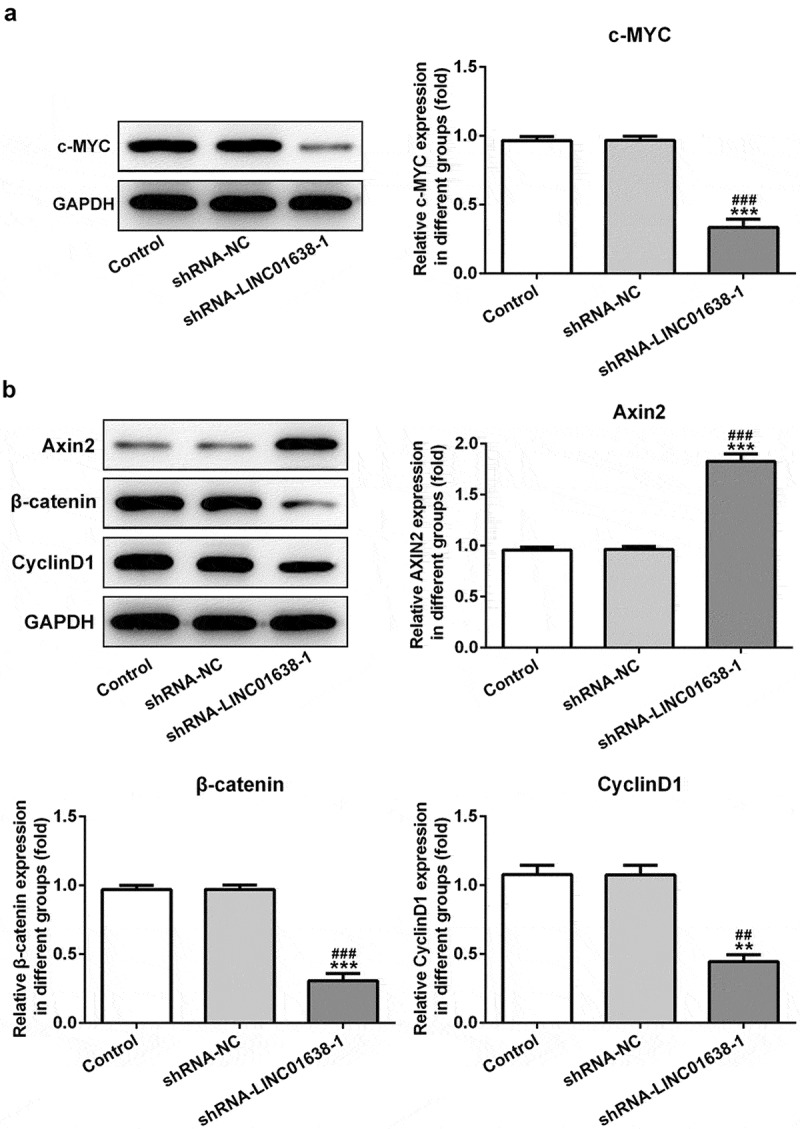
The effects of LINC01638 silencing on Wnt/β-catenin signaling pathway in TPC-1 cells. The protein level of c-MYC (a), Axin2, β-catenin and CyclinD1 (b) in TPC-1 cells with transfection of shRNA-LINC01638-1 was assessed by western blot assay. Data are expressed as mean ± SD. **P < 0.01, ***P < 0.001 versus control; ^##^P < 0.01, ^###^P < 0.001 versus shRNA-NC group

**Figure 5.**
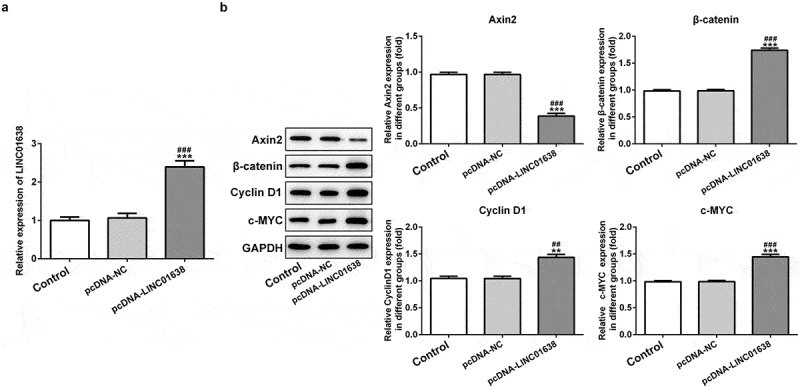
Overexpressed LINC01638 in TPC-1 cells regulates Wnt/β-catenin signaling. A, The expression of LINC01638 was measured by qRT-PCR after transfection with pcDNA-LINC01638. B, The protein expressions of c-MYC, Axin2, β-catenin and CyclinD1 were monitored by western blot analysis. Data are expressed as mean ± SD. **P < 0.01, ***P < 0.001 versus control; ^##^P < 0.01, ^###^P < 0.001 versus shRNA-NC group

We further performed the related mechanism research by which LINC01638 mediates PTC cells. We found through western blot analysis that the level of c-MYC was obviously reduced by downregulation of LINC01638 in comparison with the control cells (Figure 4a). The protein expression of Axin2 was increased and the levels of β-catenin and CyclinD1 were decreased in TPC-1 cells with transfection of shRNA-LINC01638-1 (Figure 4b). In addition, we overexpressed LINC01638 in TPC-1 cells (Figure 5a). The results showed that overexpression of LINC01638 considerably inhibited the level of Axin2 while enhanced the protein levels of c-MYC, β-catenin and CyclinD1 compared with the control and negative control groups (Figure 5b). These results indicated that LINC01638 exerted regulatory effects on PTC by c-MYC and Wnt/β-catenin.

*LINC01638 overexpression mediated TPC-1 cell proliferation, migration, invasion and cell cycle via Axin2*

**Figure 6.**
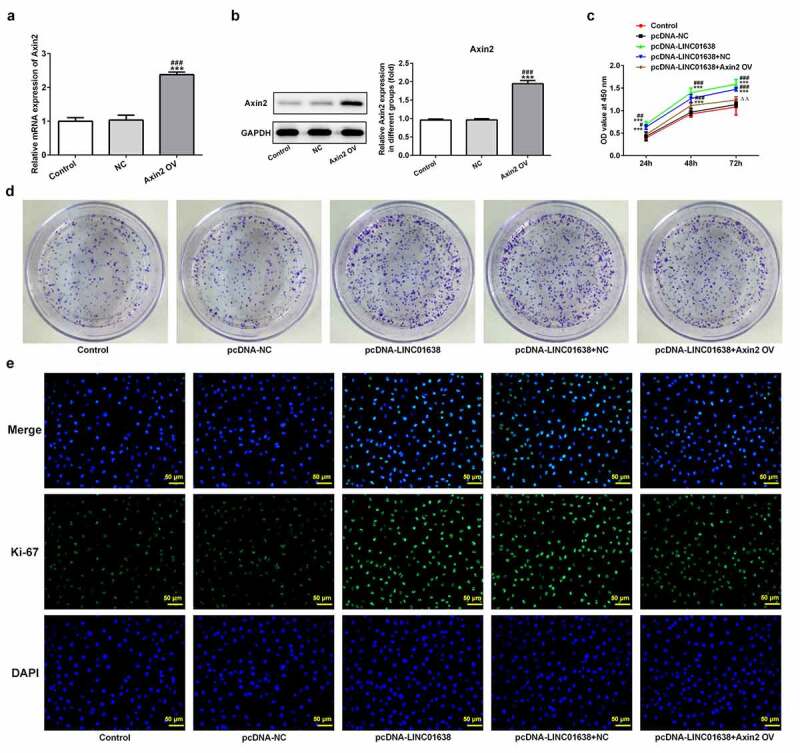
LINC01638 overexpression regulates TPC-1 cell proliferation, migration, invasion and cell cycle via Axin2. The mRNA (a) and protein expression (b) of Axin2 in TPC-1 cells transfection with Axin2-overexpressed vectors were detected by qRT-PCR and western blot assay, respectively. Cell proliferation was estimated by CCK-8 assay (c) and colony formation assay (d) after transfection with pcDNA-LINC01638 in the presence and absence of Axin2 OV. E, Immunofluorescence assay was employed to evaluate the level of Ki67 in TPC-1 cells. Data are expressed as mean ± SD. ***P < 0.001 versus control; ^#^P < 0.05, ^##^P < 0.01, ^###^P < 0.001 versus respective NC groups. ^ΔΔ^P < 0.01 versus pcDNA-LINC01638+ NC

**Figure 7.**
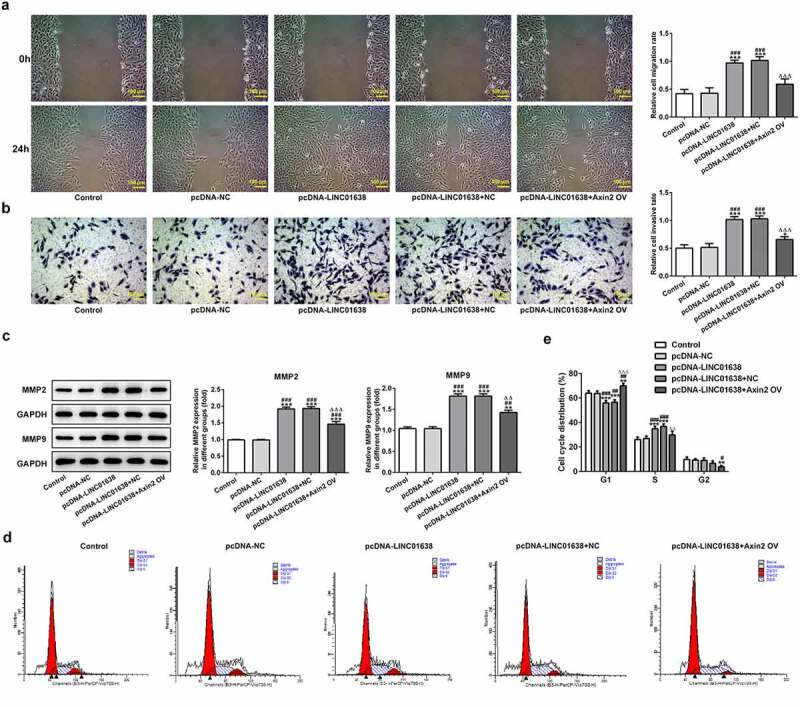
Axin2 reverses the effects of LINC01638 overexpression on PTC cell migration, invasion and cell cycle progression. A, Cell migration of TPC-1 cells was investigated by wound healing assay. B, Transwell assay was performed to determine TPC-1 cell invasion after transfection with pcDNA-LINC01638. C, The levels of MMP2 and MMP9 in TPC-1 cells transfection with pcDNA-LINC01638 in the presence and absence of Axin2 OV were measured by western blot analysis. D and E, Flow cytometric analysis was implemented to evaluate the cell cycle stage distribution. Data are expressed as mean ± SD. *P < 0.05, **P < 0.01, ***P < 0.001 versus control; ^#^P < 0.05, ^##^P < 0.01, ^###^P < 0.001 versus pcDNA-NC group. ^ΔΔ^P < 0.01, ^ΔΔΔ^P < 0.001 versus pcDNA-LINC01638+ NC

Rescue experiments were implemented to determine the influence of LINC01638 on cell proliferation, invasion and cell cycle in PTC cells. Axin2 overexpressed vectors were transfected into TPC-1 cells for upregulation of Axin2, whose expression level was detected by qRT-PCR and western blot assays (Figure 6a and 6b). CCK-8 assay and colony formation assays showed that upregulation of LINC01638 promoted the cell proliferation while Axin2 overexpression inhibited the proliferative capacity induced by LINC01638 (Figure 6c and 6d). Likewise, immunofluorescence assay revealed that the level of Ki67 was repressed by LINC01638 overexpression, and an opposite result was observed in TPC-1 cells with Axin2 overexpression (Figure 6e). Additionally, transwell assay and wound healing assay results displayed that LINC01638 overexpression obviously accelerated the migration and invasion in TPC-1 cells whereas Axin2 overexpression inhibited the ability of increased migration and invasion induced by LINC01638 overexpression (Figure 7a-7c). Moreover, transfection of pcDNA-LINC01638 brought a decrease in the G1 phase population and a notable increase in the percentage of cells in the S phase. In contrast, Axin2 overexpression reserved the effect of LINC01638 overexpression on cell cycle (Figure 7d and 7e). These data together suggested that LINC01638 was associated with the progression of PTC through the level of Axin2.

## Discussion

LncRNA LINC01638 has shown its oncogenic feature in several human cancers [15–17]. The current study revealed that lncRNA LINC01638 also acted as a tumor promoter in PTC by regulating the cell biological process. The influence of LINC01638 on PTC cells may result from the altered expressions of Wnt/β-catenin signaling pathway. The expression levels of a series of lncRNAs are altered in the origination and development of PTC [18]. The changed levels of lncRNAs imply the stage and severity of the diseases, pointing to the feasibility of the use of them for disease diagnosis, therapy and prognosis [19]. Many studies have showed that upregulation or downregulation of some certain lncRNAs in cancers can regulate the progression of the cancers [20]. Lei et al. demonstrated that LncRNA Taurine up-regulated gene 1 (TUG1) regulated the proliferative and migrative processes of PTC cells and reversed EMT formation through sponging miR-145 [21]. Zhang et al. revealed that LncRNA Gas5 was significantly downregulated in both PTC tissues and cell lines. Upregulation of Gas5 dramatically inhibited the cell proliferation and the tumor growth in PTC by the activation of PTEN/AKT pathway via sponging miR-222-3p [22]. Liu et al. reported that knockdown of lncRNA DUXAP8 inhibited the proliferation, migration and invasion of PTC cells, revealing that lncRNA DUXAP8 acted as an oncogene in PTC [23]. In the present study, LINC01638 expression in several PTC cells was reported to be higher than the thyroid gland epithelial cells. LINC01638 silencing played its tumor suppressive role in PTC by inhibiting cell growth and disturbing the cell cycle.

E26 transformation-specific (ETS) factors have been regarded as the principal end-effectors of the BRAF-ERK signaling cascade, implicated in thyroid carcinogenesis [24,25]. ELK1, one member of ETS factors, was also reported to interact with thyroid relevant gene promoters, presenting a regulatory role in thyroid cancer [26]. Through JASPAR software prediction, we found that ELK1 was one transcription factor of LINC01638, so the interaction between LINC01638 and ELK1 raised our interest. The luciferase reporter assay and gain- and loss-functional experiments demonstrated the binding relationship between LINC01638 and ELK1, indicating that ELK1 bound to LINC01638 promoter to regulate the expression level of LINC01638, thus participating to and regulating PTC progression.

c-Myc is the gene product of MYC, an oncogene that can facilitate tumorigenesis in many human tumors [27]. c-Myc has been proved to be associated with tumor aggression and poor clinical outcome [28]. Studies showed that LINC01638 was highly expressed in triple-negative breast cancer (TNBC) tissues and LINC01638 mediated TNBC progression by interacting with c-Myc and activating MTDH-Twist1 signaling [13]. Another study demonstrated a high level of c-Myc in PTC tissues and cells. Silencing of LIN28A can mute the expression of the downstream gene c-Myc, thus slowing down PTC cell proliferation, migration and invasion [29]. Besides, Wnt/β-catenin pathway as the upstream signaling pathway of c-Myc has already been found to be an essential participant in PTC [30–33]. Wang et al. revealed that LncRNA PTCSC3 and miR-574-5p mediated the proliferation and migration of PTC cells via targeting SCAI and regulating the activation of Wnt/β-catenin [34]. Ding et al. reported that LncRNA SNHG12 was involved in cell proliferation, cell cycle and metastasis of PTC through altering the protein expressions of Wnt/β-catenin signaling pathway [35]. In our study, ELK1 was found to be the transcription factor of LINC01638. The Wnt/β-catenin pathway is a bridge between the functions of ELK1 and lung adenocarcinoma that can be regulated by ELK1 [36]. We further noticed that β-catenin, Cyclin D1 and c-MYC levels in Wnt/β-catenin pathway were inhibited upon knockdown of LINC01638 and were increased upon overexpression of LINC01638. Of note, Cyclin D1 is an important regulator of cell cycle progression during G1 phase, an event essential in G1-S transition, exerting multiple functions in cell biology, including cell proliferation, growth regulation and migration control [37]. In addition, high cyclin D1 expression level was also observed in patients with recurrence/distant metastasis with PTC, suggesting an involvement of cyclin D1 in PTC and a potential oncogenic activity of cyclin D1 [38]. These results clarified that ELK1-induced high level of LINC01638 boosted the proliferation, migration and invasion of PTC cells partly through regulating Wnt/β-catenin signaling-mediated cell cycle progression.

With this in mind, we speculated that LINC01638 silencing inactivated Wnt/β-catenin signaling via exciting the Axin2. Thus, we performed the reverse experiment base on the above results. The present study revealed that overexpressed LINC01638 significantly inhibited the expression of Axin2 while enhanced the levels of β-catenin, CyclinD1 and c-MYC in TPC-1 cells. In addition, Axin2 takes part in the composition and is identified as negative regulator of the canonical Wnt signaling pathway [39]. In the current study, Axin2 was overexpressed by transfection with Axin2-overexpressed vectors and the results showed that upregulation of Axin2 inhibited PTC cell proliferation, repressed its migration and invasion and accelerated cell cycle progression. These results suggested that LINC01638 modulated the biological process of PTC cells via regulation of Axin2.

## Conclusions

In conclusion, we showed for the first time that LINC01638 is the potential oncogene in PTC. LINC01638 expression was remarkably upregulated in PTC cells and ELK1-induced upregulation of LINC01638 promoted the proliferation, accelerated the metastasis and strengthened the cell cycle of PTC cells. Moreover, LINC01638 may exert its regulatory effects on PTC through Wnt/β-catenin signaling via negative regulation of Axin2. This study provides a novel perspective on the treatment and favorable prognosis of patients with PTC.

## Data Availability

All data generated or analyzed during this study are included in this published article.
